# Field Evaluation of Selected Plant Volatiles and Conspecific Pheromones as Attractants for *Agriotes obscurus* and *A. lineatus* (*Coleoptera: Elateridae*)

**DOI:** 10.3390/insects13020173

**Published:** 2022-02-06

**Authors:** Wim van Herk, Bob Vernon, Gabrielle Bourassa-Tait, Miklós Tóth, Ervin Kovacs

**Affiliations:** 1 Agassiz Research and Development Centre, Agriculture and Agri-Food Canada, P.O. Box 1000, Agassiz, BC V0M 1A0, Canada; gabrielle.bourassa-tait@agr.gc.ca; 2 Sentinel IPM Services, 4430 Estate Drive, Chilliwack, BC V2R 3B5, Canada; boomervernon@msn.com; 3 Plant Protection Institute, Centre for Agricultural Research, Pf. 102, H-1525 Budapest, Hungary; toth.miklos@atk.hu; 4 Intko Supply Ltd., Suite 428, 8-6014 Vedder Rd., Chilliwack, BC V2R 5P5, Canada; pheromonesupply@gmail.com

**Keywords:** click beetles, wireworms, semiochemicals, integrated pest management, trapping

## Abstract

**Simple Summary:**

Wireworms are the larval stage of click beetles, and are serious pests of multiple field crops worldwide. Pitfall traps for collecting the adult (beetle) stage of these pests are commonly used to determine the risk of crop injury, or to remove beetles from a field before they mate or lay eggs, but such traps are only effective when baited with an attractant. These attractants are typically sex pheromones produced by female beetles and attractive to males, but two blends of plant-derived compounds attract females of some species. We evaluated the attractiveness of these compounds to male and female *Agriotes obscurus* and *A. lineatus*, species that are pests in both Europe and North America. However, both blends appeared to reduce captures, even when combined with the pheromones of these species. Similarly, combining the pheromones of these two beetles into a single lure reduced captures of both species. Our results indicate that attractants for female click beetles may be highly species-specific, and that combining pheromones of closely related species with each other or with plant volatiles can reduce trapping efficacy. The development of attractants for females of key pest species would greatly advance our ability to monitor and manage these pests.

**Abstract:**

Sex pheromones are commonly used in traps to monitor populations and movements of male click beetles, but to date few attractants have been identified for females. Notable exceptions are plant-derived kairomones for female *Agriotes brevis* and *A. ustulatus*, allowing the monitoring of both males and females of these species with lures containing both pheromones and plant volatiles. The attractiveness of these plant volatiles for two congeners, *A. obscurus* and *A. lineatus*, which are agricultural pests in Europe and North America, was evaluated in the current study. Both the four-component MINIM plant-derived lure for *A. brevis*, and the blend of (*E*)-anethol and (*E*)-cinnamaldehyde for *A. ustulatus*, were not attractive to *A. obscurus* and *A. lineatus*, and instead appeared to reduce captures—both when compared to blank controls, and when blended with and compared to the sex pheromones of these species. This was most pronounced in *A. obscurus*, where (*E*)-anethol and (*E*)-cinnamaldehyde reduced male captures by 43 and 37%, respectively. Combining the pheromones of *A. obscurus* and *A. lineatus* reduced captures of these species by 77 and 19%, respectively, compared to these pheromones singly. This suggests that attractants for female click beetles can be highly species-specific, and that the blending of pheromones of congeneric species with each other, or with plant volatiles, can reduce captures. Further research into developing such attractants for economic species is urgently needed.

## 1. Introduction

Wireworms, the larval form of pestiferous click beetle species (*Coleoptera: Elateridae*), are increasingly important pests of cereals, corn, potato, vegetables and other field crops worldwide [1,2,3,4]. Due to their subterranean nature, patchy distributions and seasonal vertical movements in the soil, wireworm abundance in agricultural land is generally hard to assess using soil coring or bait trapping methods for larvae, and the presence and relative abundance of pest species can be determined more easily with traps attractive to beetles [1,5]. Recent work has demonstrated that pheromone-baited traps for click beetles can be used to assess the risk of crop damage from wireworms [6,7,8,9], and also that sex pheromones can be used in mating disruption, mass trapping, and attract-and-kill strategies for reducing pest populations [5,10,11,12,13]. At present, all click beetle pheromones identified are female-produced and attractive primarily to males. The availability of semiochemical attractants for female beetles would also be beneficial in determining how and when they enter and/or disperse across fields, when to apply management tactics (e.g., application of chemical sprays or biopesticides prior to oviposition) [1,14], or if management tactics are needed at all (e.g., if populations are low) [15].

To date, very few attractants for female click beetles are known. Observations of female *Agriotes brevis* Candeze aggregating beneath the freshly cut foliage of *Medicago sativa* (L.) and *Lolium italicum* (A. Br.) placed on plastic sheets on the soil, and subsequent analyses of volatile extracts and antennal responses of *A. brevis* to these extracts, led to the development of a four-component “MINIM” plant-derived lure attractive to female *A. brevis* in the field [16]. Observations of bycatch of *A. ustulatus* Schaller on traps deployed for western corn rootworm (*Diabrotica v. virgifera* Le Conte) and other insects led to the development of a blend of (*E*)-anethol and (*E*)-cinnamaldehyde, two floral compounds, which is attractive to both male and female *A. ustulatus* [17]. In addition, females of at least three click beetle species, *A. sordidus* (Illiger), *A. brevis*, and *A. ustulatus*, are attracted to their own pheromones [18,19], though not as strongly as conspecific males. These observed behaviors have allowed for the development of lures that are attractive to both sexes of a species. For example, traps baited with a combination of pheromone and plant-derived lures for *A. ustulatus* resulted in higher catches than those baited with either compound alone [20].

The objective of the current study was to determine whether male and female *A. obscurus* L. and *A. lineatus* L., two species of economic importance in both North America and Europe [5], are attracted to the plant volatile blends attractive to *A. brevis* and *A. ustulatus*. Beetles were exposed to these blends singly, and in combination with their own or the heterospecific’s pheromone.

## 2. Materials and Methods

Two studies were conducted. To determine the attractiveness of the MINIM lureand/or AO pheromone to male and female *A. obscurus* (AO), Vernon Beetle Traps [21] were deployed in a recently plowed and disked 4 ha field that had previously been in long-term pasture at the Agassiz Research and Development Centre (ARDC) in Agassiz, BC, Canada, in 2013 (49.250338°N, 121.766312°W). Traps were baited with one or both compounds in separate lures, or left unbaited (control traps). MINIM lures (Plant Protection Institute, Centre for Agricultural Research, Budapest, Hungary) contained a blend of (*Z*)-3-hexenyl acetate (300 mg), methyl benzoate (5 mg), (*Z*)-3-hexenol (30 mg), and methyl salicylate (30 mg), and AO pheromone lures (Csalomon^®^ Plant Protection Institute, Centre for Agricultural Research, Budapest, Hungary) contained 175 μL of a blend of geranyl hexanoate and geranyl octanoate (1:1 ratio). The traps were placed during the peak swarming season (26 April) in three side-by-side 4 × 4 Latin square blocks with 8 m spacing between traps and 16 m between blocks, to effectively form a single 4-treatment, 12-replicate RCBD study. Care was taken to prevent cross-contamination between traps during trap checks (3, 17 May), by using different gloves for different treatments during trap installation and checks. All beetles—AO, and incidental catches of co-occurring *A. lineatus* (AL)—were identified by species and their sexes were determined.

To determine the attractiveness of a blend of (*E*)-anethol and (*E*)-cinnamaldehyde (hereafter “plant volatiles”) to male and female AO and AL, Vernon Pitfall Traps^TM^ (VPT, Intko Supply Ltd., Chilliwack, BC, Canada) [22] were deployed in a long-term blueberry field at ARDC in 2021 (49.243518°N, 121.756290°W). The traps were baited with the plant volatiles and/or the AO (60 μL, 1:1 geranyl hexanoate:geranyl octanoate) or AL (40 μL, 1:1 geranyl butanoate:geranyl octanoate) pheromone (combined in single lures), were left unbaited (control traps), or were baited with a lure containing both the 60 μL AO and the 40 μL AL pheromone. The purpose of the latter treatment was to confirm if combining the two pheromones reduced captures of AO beetles, as observed by Vernon et al. [23]. All lures were capsule lures obtained from Intko Supply. Traps were placed during the peak swarming season (10 May), 8 m apart in 6 parallel rows, with rows also 8 m apart and running between or parallel to the blueberry rows, thus effectively making a single 7-treatment, 12-replicate RCBD study. As 28 (of 84) traps were located outside of the outermost blueberry plants in the plot, and a further 8 traps were near the ends of the blueberry rows, three landscape categories were created (Outside-Plots and Row-End, and Main for the 48 remaining traps) to determine if the proximity of the blueberry plants to the traps affected trap captures. All traps were located in identical and uniform vegetation consisting of short lawn grass mowed shortly before the study commenced, and placement in these landscape types was random. Trap checks were conducted on 12, 20 and 27 May, and care was taken to prevent cross-contamination between traps during trap installation and checks, as above. Pheromone capsule lures were left open to allow for the free diffusion of volatiles [22,24]. All beetles were identified to species, but due to the large number collected in 2021, the sexes of only 40 AO and 40 AL beetles were determined per trap for each collection date (i.e., all beetles per trap if ≤40), except for five treatments in the first collection, where the sex was determined for all beetles.

### Statistical Analysis

The numbers of male and female beetles collected in different treatments were analyzed per sex (in 2013) or combined (in 2021) using generalized linear models (GLM) (Proc GENMOD, SAS 9.2, SAS Institute, Cary, NC, USA), with a negative binomial distribution and log-link function. For the 2021 data, the proportions of female beetles in the subsamples sexed per trap were analyzed with a GLM with a binomial distribution and a logit-link function. For the 2021 data, the treatment replicate was nested within the landscape category. Where significant differences among treatments were observed, treatment means were separated using Tukey’s Standardized Range HSD test at *p* = 0.05.

## 3. Results and Discussion

### 3.1. Response of AO and AL to MINIM

Traps baited with AO pheromone collected significantly more male AO beetles than traps baited with MINIM lures or left unbaited on both collection dates, but traps with MINIM lures attracted numerically fewer male AO than control traps (Table 1). Baiting with both MINIM and the AO pheromone numerically reduced catches of males relative to the pheromone-only treatment (8% overall, Table 1), but did not significantly deter male AO beetles from trap entry. It is interesting that significantly more AO females were collected in traps baited with AO pheromone (alone) than in control traps on 3 May and in total (Table 1), indicating some attraction. Pheromone autodetection by female beetles has been reported previously in *Agriotes* [18,19], and has been observed for AO before (RSV, unpublished data), suggesting that some click beetle pheromones may also function as aggregation cues [19]. The reduced attractiveness of the AO pheromone to female AO on 17 May corroborates previous observations that such attractiveness wanes over the season (RSV, unpublished data), potentially due to changes in their mating status. It is possible that female AO attraction to their own pheromone increases the likelihood of mating, e.g., by aggregated females collectively producing a stronger pheromone plume for better detection by males. Incidentally, pheromone autodetection has been observed in other elaterid genera, but with females being repelled by their own pheromone [25]. As with males, fewer (9% overall, Table 1) female AO were collected in traps baited with the MINIM lures than in unbaited traps, suggesting this compound is not attractive to AO.

Traps baited with AO pheromone also collected a significant number of male *A. lineatus* in the 17 May but not in the 3 May collection (Table 1). This confirms that male AL are somewhat attracted to the AO pheromone, as noted in a study by Vernon et al. [26], where nearly 30% of mark-released AL males were caught in AO pheromone-baited traps in the absence of AL pheromone traps, and that the seasonal emergence of AL in western Canada is typically 2 weeks later than that of AO [27]. The similar numbers of male AL collected in the AO (alone) and AO + MINIM-baited traps (Table 1) suggest this species is not deterred by the latter compound

### 3.2. Response of AO and AL to (E)-anethol and (E)-cinnamaldehyde

Traps baited with the plant volatiles collected fewer AO beetles than unbaited control traps on all three dates (43% overall), but this difference was only statistically significant for the 27 May and combined collections (Table 2). For each date, the proportion of females was numerically higher in traps baited with plant volatiles, suggesting male beetles were more deterred than females. Traps baited with AL pheromone also collected significantly fewer AO beetles than control traps on 20 and 27 May (40% overall), corroborating earlier data [23] that AO beetles are repelled by the AL pheromone (Table 2). There was no significant difference in the proportion of female AO beetles between these three treatments for 12, 20 and 27 May, but the high proportion (0.73, Table 2) in the AL pheromone-baited traps relative to the control (0.35) on 27 May suggests male AO deterrence may increase over time. The proportion of female AO in the unbaited control traps increased from 0.05 to 0.25 to 0.35 during this study (Table 2), indicating that the abundance and/or activity of female AO increased over time relative to that of males.

As expected, the highest number of AO were collected in traps baited with AO pheromone (Table 2). Combining the plant-derived compounds with the AO pheromone reduced captures by 37, 32, and 42% for the 12, 20, and 27 May collections, respectively (37% overall), with the reduction statistically significant for 27 May (Table 2). The numerically higher proportion of females in the combined treatment vs. AO pheromone alone for this date (0.12 vs. 0.06, Table 2) again may suggest male beetles are more repelled by the plant volatiles than females.

Combining the AO and AL pheromone into a single lure reduced captures of AO beetles by 94, 79, and 67% for the 12, 20, and 27 May collections, respectively (77% overall), with the reduction statistically significant for each date (Table 2). The relatively high proportion of females in the 20 May and combined collections (0.10 vs. 0.03, 0.07 vs. 0.04, respectively, AO + AL vs. AO treatments, Table 2) suggests this reduction is driven primarily by lower captures of males. This result is similar to earlier findings by Vernon et al. [23], where combining constituents of AO and AL pheromones into a single lure (1:1:1 geranyl butanoate: geranyl hexanoate: geranyl octanoate) reduced AO captures by 76% (mean of 19 field sites), and indicates male AO are repelled by geranyl butanoate (a constituent in AL but not AO pheromone).

Traps baited with the plant volatiles collected fewer AL beetles than unbaited control traps overall (17%), but this reduction was only observed on the 12 May and in combined collections (Table 2), and was far less pronounced than for AO. Also unlike AO, where the pheromone of AL reduced captures, traps baited with AO pheromone collected more AL beetles on each date (47%, overall) than control traps, again corroborating that AL beetles are attracted to the AO pheromone (Table 2).

As expected, the highest number of AL were collected in traps baited with AL pheromone (Table 2). Similar to AO, combining the plant volatiles with the AL pheromone reduced AL captures by 12, 45, and 56% on 12, 20, and 27 May, respectively (41% overall), relative to the AL pheromone alone, with the reduction statistically significant for the 20 and 27 May and combined collections (Table 2). Traps baited with AO pheromone and plant volatiles captured 20, 73, and 50% fewer AL on the 12, 20, and 27 May collections, respectively (34% overall), than traps baited with AO pheromone alone, again suggesting the plant volatiles repelled AL beetles from entering traps.

Combining the AO and AL pheromone into a single lure increased captures of AL beetles by 10% in the 12 May collection, but reduced captures by 23 and 36% for the 20 and 27 May collections, respectively (19% overall); none of these differences were statistically significant (Table 2). This result is intriguing as it may suggest that the attractiveness of AO pheromones to AL beetles changes over time, possibly as a function of beetle age or mating status. It also contradicts the findings of Vernon et al. [23], where combining constituents of AO and AL pheromones into a single lure (noted above) increased AL captures by 42% (mean of 19 field sites), but this discrepancy may be due to the higher ratio of geranyl octanoate in the current lures (i.e., 1:1:2, respectively). The reduction in beetle captures resulting from combining the various compounds reported herein is not thought to result from interactions between the compounds or reduced diffusion rates, as captures were also lower when plant compounds were tested alone, and as capsule lids (in 2021) were kept open throughout the study.

### 3.3. Implications and Research Needs

Unlike in Europe, where pheromones for most of the economically important click beetle species have been identified and used in monitoring for some time [28,29,30,31,32,33], work on identifying sex pheromones for key North American pest species has only recently resumed [25,34,35,36]. As in Europe, these pheromones are typically used for monitoring the populations and distributions of male beetles [24,37,38,39], but provide little if any information on the activity periods and movements of female beetles across agricultural landscapes. Hence, considerable work is urgently needed, if possible, to develop lures that attract female beetles. From the work discussed herein, it appears that such lures may be highly species-specific, and should rely on observations of the preferred host plants and/or oviposition sites in fields of different species. Ideally, candidate compounds would then be identified with GC-EAD using the antennae of both male and female beetles, followed with olfactometry lab bioassays and the subsequent field testing of putative attractants. The development of female-specific lures may also enable us to monitor, and possibly manage, North American pest species that are (mostly) parthenogenetic, such as *Hypnoidus bicolor* (Eschscholtz) and *Aeolus mellillus* Say [1].

Provided that the compounds are not repellent to either sex, click beetle pheromones and plant volatiles can potentially be combined to produce lures that are more attractive than those containing either compound singly, as demonstrated for *A. brevis* and *A. ustulatus* [19,20]. Similarly, sex pheromones can be combined into single lures to collect multiple pest species, as has been done for *A. lineatus* and various *Limonius* species in BC (WvH, unpublished data). When pheromone lures for AL and AO were combined, no difference was found in catches of AL in a preliminary study in Europe [40], which led to the suggestion of using both lures together in the same trap. However, when pheromones for these species were combined in Canada, captures of the former species were reduced [23], and as shown herein, captures of AL and AO may also be reduced when their pheromones are combined with non-attractive plant volatiles. This suggests that the combination possibilities of different pheromonal or plant-derived lures of click beetles require in-depth study in the future.

Finally, the development of plant-derived or pheromone lures should also take into consideration that a species’ response to an attractant may vary with geographic region, possibly because of differences in pheromone composition [41] and in their relative haplotypes abundance between locations [42].

## 4. Conclusions

Although attractive to some *Agriotes* species, the plant-derived compound blends evaluated in these studies were not attractive to male or female AO and AL, but instead appeared to reduce captures. This suggests that the existence and nature of attractants for female click beetles will need to be determined per species, and that considerably more research is needed to determine whether pest species have preferred host plants for food sources and/or oviposition sites. AL beetles are attracted to the AO sex pheromone, but not vice versa, and combining the pheromones of these two species can reduce captures of both. While blending these pheromones in a single lure may allow the detection of both species in a field, it will reduce the effectiveness of traps baited with such lures for mass trapping.

## Figures and Tables

**Table 1:** Capture of *Agriotes obscurus* (AO) and *A. lineatus* (AL) male and female click beetles in traps baited with MINIM, a plant-derived attractant to *A. brevis*, and/or *A. obscurus* pheromone, in 2013. Shown are mean (SEM) numbers from 12 replicates. *n* = total beetles. For each species, numbers in columns followed by the same letter are not significantly different (α = 0.05; Tukey’s HSD). The AL beetles were incidental bycatches in this study, which was principally designed to attract AO.

	3 May		17 May		Total (Dates Combined)	
*Agriotes obscurus*						
Trap Lure	Male	Female	Male	Female	Male	Female
MINIM	1.6 (0.34) B	2.9 (0.67) C	1.3 (0.36) B	3.4 (0.77) B	2.9 (0.51) B	6.2 (1.39) C
MINIM + AO pheromone	14.7 (3.62) A	7.4 (1.09) AB	12.8 (3.16) A	7.7 (1.48) A	27.4 (6.54) A	15.1 (2.19) AB
AO pheromone	16.5 (1.62) A	9.6 (1.86) A	13.3 (2.75) A	7.0 (1.09) A	29.8 (4.08) A	16.6 (2.44) A
Blank control	2.7 (0.61) B	4.5 (0.94) BC	1.7 (0.47) B	5.3 (0.74) AB	4.5 (0.72) B	9.9 (1.68) BC
*N*	425	293	346	271	768	557
Treatment (df = 3)	Chi = 57.3, *p* < 0.0001	Chi = 17.5, *p* = 0.0006	Chi = 50.1, *p* < 0.0001	Chi = 10.2, *p* = 0.017	Chi = 66.9, *p* < 0.0001	Chi = 16.4, *p* = 0.001
Replicate (df = 11)	Chi = 25.7, *p* = 0.007	Chi = 11.9, *p* = 0.38	Chi = 37.7, *p* < 0.0001	Chi = 17.7, *p* = 0.09	Chi = 41.9, *p* < 0.0001	Chi = 16.1, *p* = 0.14
*Agriotes lineatus*						
Trap Lure	Male *	Female *	Male	Female *	Male	Female *
MINIM	0.0 (0.00)	0.0 (0.00)	0.0 (0.00) A	0.0 (0.00)	0.0 (0.00) A	0.0 (0.00)
MINIM + AO pheromone	0.4 (0.19)	0.1 (0.08)	13.3 (1.29) B	0.2 (0.11)	13.8 (1.36) B	0.3 (0.13)
AO pheromone	0.4 (0.19)	0.0 (0.00)	11.6 (0.67) B	0.0 (0.00)	12.0 (0.70) B	0.0 (0.00)
Blank control	0.0 (0.00)	0.0 (0.00)	0.0 (0.00) A	0.1 (0.09)	0.0 (0.00) A	0.1 (0.09)
*N*	10	1	299	3	309	4
Treatment (df = 3)			Chi = 122.9, *p* < 0.0001		Chi = 122.1, *p* < 0.0001	
Replicate (df = 11)			Chi = 15.8, *p* = 0.15		Chi = 18.0, *p* = 0.08	

* numbers too low for statistical analyses.

**Table 2:** Captures of male (M) and female (F) *Agriotes obscurus* (AO) and *A. lineatus* (AL) click beetles in traps baited with AO or AL sex pheromone (ph) and/or (*E*)-anethol and (*E*)-cinnamaldehyde, plant volatiles (PV) attractive to *A. ustulatus*, in 2021. Shown are mean (SEM) captures from 12 replicates. Prop. F = proportion female beetles from subsamples sexed per trap. *n* = number of beetles collected (M + F column), or number of females out of number that were sexed (Prop. F column). For each species, numbers in columns followed by the same letter are not significantly different (α = 0.05; Tukey’s HSD).

	12 May		20 May		27 May		Total (dates combined)	
*Agriotes obscurus*								
Trap Lure	Total (M + F)	Prop. F	Total (M + F)	Prop. F	Total (M + F)	Prop. F	Total (M + F)	Prop. F
Blank control	7.0 (1.63) BC	0.05 (0.03) A	11.5 (3.29) BC	0.25 (0.06) A	5.4 (0.91) D	0.35 (0.07) AB	23.9 (4.72) BC	0.22 (0.04) AB
PV	2.8 (1.08) CD	0.12 (0.06) A	8.1 (2.76) C	0.37 (0.09) A	2.6 (0.61) E	0.44 (0.10) A	13.5 (3.66) D	0.37 (0.06) A
AO ph	15.6 (2.63) A	0.03 (0.01) A	103.9 (20.36) A	0.03 (0.01) C	42.6 (7.27) A	0.06 (0.02) C	162.1 (28.07) A	0.04 (0.01) C
AO ph + PV	9.8 (2.20) AB	0.00 (0.00) A	70.2 (16.60) A	0.05 (0.02) C	22.5 (3.38) B	0.12 (0.03) C	102.5 (21.30) A	0.07 (0.02) C
AL ph	11.1 (2.82) AB	0.02 (0.02) A	2.4 (0.62) D	0.26 (0.13) AB	0.8 (0.32) F	0.73 (0.19) A	14.3 (3.26) CD	0.09 (0.03) BC
AL ph + PV	2.0 (1.17) D	0.08 (0.08) A	2.7 (0.79) D	0.32 (0.13) A	0.3 (0.13) F	0.67 (0.33) ABC	4.9 (1.66) E	0.32 (0.11) A
AO ph + AL ph	1.0 (0.30) D	0.00 (0.00) A	21.8 (4.69) B	0.10 (0.04) BC	13.9 (3.10) C	0.04 (0.03) C	36.8 (6.53) B	0.07 (0.03) C
*n*	592	18 (592)	2647	108 (1414)	1057	93 (922)	4296	219 (2928)
Treatment (df = 6)	Chi = 47.7, *p* < 0.0001	Chi = 18.2, *p* = 0.006	Chi = 108.7, *p* < 0.0001	Chi = 118.1, *p* < 0.0001	Chi = 135.1, *p* < 0.0001	Chi = 100.2, *p* < 0.0001	Chi = 103.4, *p* < 0.0001	Chi = 160.8, *p* < 0.0001
Replicate (df = 19)	Chi = 28.4, *p* = 0.08	Chi = 17.7, *p* = 0.54	Chi = 69.6, *p* < 0.0001	Chi = 58.3, *p* < 0.0001	Chi = 53.5, *p* < 0.0001	Chi = 60.3, *p* < 0.0001	Chi = 57.7, *p* < 0.0001	Chi = 92.5, *p* < 0.0001
*Agriotes lineatus*								
Trap Lure	Total (M + F)	Prop. F^1^	Total (M + F)	Prop. F^1^	Total (M + F)	Prop. F^1^	Total (M + F)	Prop. F^1^
Blank control	2.3 (0.57) B	0.00 (0.00)	1.1 (0.45) C	0.20 (0.16)	0.3 (0.13) C	0.00 (0.00)	3.6 (0.73) C	0.03 (0.02)
PV	1.2 (0.65) B	0.00 (0.00)	1.3 (0.43) C	0.00 (0.00)	0.6 (0.23) C	0.20 (0.20)	3.0 (0.92) C	0.03 (0.03)
AO ph	3.2 (1.05) B	0.00 (0.00)	1.5 (0.44) C	0.12 (0.08)	0.6 (0.23) C	0.10 (0.10)	5.3 (1.29) C	0.06 (0.03)
AO ph + PV	2.8 (0.78) B	0.00 (0.00)	0.4 (0.23) C	0.33 (0.33)	0.3 (0.14) C	0.25 (0.25)	3.5 (0.99) C	0.06 (0.06)
AL ph	61.3 (11.91) A	0.00 (0.00)	333.4 (73.63) A	0.00 (0.00)	33.8 (9.59) A	0.02 (0.01)	428.5 (91.30) A	0.00 (0.00)
AL ph + PV	53.8 (7.04) A	0.00 (0.00)	183.6 (27.22) B	0.00 (0.00)	14.8 (3.88) B	0.00 (0.00)	252.2 (36.38) B	0.00 (0.00)
AO ph + AL ph	67.4 (10.11) A	0.00 (0.00)	256.0 (40.36) AB	0.00 (0.00)	21.7 (5.07) AB	0.00 (0.00)	345.1 (52.43) AB	0.00 (0.00)
*n*	2301	0 (1784)	9327	5 (1491)	865	7 (726)	12493	12 (4001)
Treatment (df = 6)	Chi = 137.2, *p* < 0.0001		Chi = 177.2, *p* < 0.0001		Chi = 113.2, *p* < 0.0001		Chi = 205.9, *p* < 0.0001	
Replicate (df = 19)	Chi = 51.1, *p* < 0.0001		Chi = 84.2, *p* < 0.0001		Chi = 62.1, *p* < 0.0001		Chi = 77.6, *p* < 0.0001	

^1^ numbers too low for statistical analyses.

## Data Availability

The data presented in this study are available on request from the corresponding author.
